# No evidence for copy number and methylation variation in *H19* and *KCNQ10T1* imprinting control regions in children born small for gestational age

**DOI:** 10.1186/1471-2350-15-67

**Published:** 2014-06-17

**Authors:** Rinki Murphy, John MD Thompson, Jörg Tost, Edwin A Mitchell

**Affiliations:** 1 Department of Medicine, Faculty of Medical and Health Sciences, University of Auckland, Private Bag 92019, Auckland, New Zealand; 2 Department of Paediatrics, Faculty of Medical and Health Sciences, University of Auckland, Auckland, New Zealand; 3 Laboratory for Epigenetics and Environment, Centre National de Genotypage, CEA-Institut de Genomique, Evry, France

**Keywords:** DNA methylation, Imprinting, ICR2, ICR1, H19, KCNQ10T1, Small for gestational age

## Abstract

**Background:**

There is a substantial genetic component for birthweight variation, and although there are known associations between fetal genotype and birthweight, the role of common epigenetic variation in influencing the risk for small for gestational age (SGA) is unknown. The two imprinting control regions (ICRs) located on chromosome 11p15.5, involved in the overgrowth disorder Beckwith-Wiedemann syndrome (BWS) and the growth restriction disorder Silver-Russell syndrome (SRS), are prime epigenetic candidates for regulating fetal growth. We investigated whether common variation in copy number in the BWS/SRS 11p15 region or altered methylation levels at *IGF2/H19* ICR or *KCNQ10T1* ICR was associated with SGA.

**Methods:**

We used a methylation-specific multiplex-ligation-dependent probe amplification assay to analyse copy number variation in the 11p15 region and methylation of *IGF2*/*H19* and *KCNQ10T1* ICRs in blood samples from 153 children (including 80 SGA), as well as bisulfite pyrosequencing to measure methylation at *IGF2* differentially methylated region (DMR)0 and *H19* DMR.

**Results:**

No copy number variants were detected in the analyzed cohort. Children born SGA had 2.7% lower methylation at the *IGF2* DMR0. No methylation differences were detected at the *H19* or *KCNQ10T1* DMRs.

**Conclusions:**

We confirm that a small hypomethylation of the *IGF2* DMR0 is detected in peripheral blood leucocytes of children born SGA at term. Copy number variation within the 11p15 BWS/SRS region is not an important cause of non-syndromic SGA at term.

## Background

Term infants born small for gestational age (SGA) have high rates of perinatal morbidity and mortality [1] as well as increased risk of later-life chronic diseases such as type 2 diabetes, cardiovascular disease, obesity and hypertension [2]. Birth weight variation has a significant genetic component with clustering of SGA births in families and recurrence in successive generations [3,4], and heritability estimates from family and twin studies range between 25-40% [5]. Recent data from genome wide association studies have found robust associations between fetal genotype and birth weight [6], and we have previously demonstrated that genetic variation in certain genes associated with obesity and or type 2 diabetes is more prevalent in those born SGA than those born appropriate for gestational age (AGA) [7]. Currently, little is known about the role of epigenetic modifications and copy number variation in determining the risk of SGA.

The term epigenetics refers to heritable changes in gene expression that are not encoded by alterations within the DNA sequence and includes, non-coding RNAs, methylation of DNA and a range of histone modifications. The *IGF2/H19* and *KCNQ10T1* imprinting control regions (ICRs) are strong candidates for the epigenetic influence on birth weight variation as they are implicated in the neonatal overgrowth and growth restriction conditions of Beckwith Wiedemann (BWS) and Silver-Russell (SRS) syndromes, respectively. Severe hypomethylation of the *KCNQ10T1* ICR [8] and hypermethylation of the *H19* DMR is found in 50% and 10% of BWS cases, respectively [9]. Conversely, severe hypomethylation of the *H19* DMR or *IGF2* DMR on the paternal allele (associated with *IGF2* repression) is found in over 50% of patients with Silver-Russell Syndrome [10], characterized by intrauterine and post-natal growth retardation [11]. Copy number variation through uniparental disomy and small deletions or duplications affecting both these regulatory sites are also recognised causes of BWS and SRS [12]. Maternal duplication of this region is associated with growth retardation [13,14], while paternal duplication is associated with overgrowth at birth [8].

The *IGF2/H19* ICR contains several differentially methylated regions (DMRs), which are all predominantly methylated on the paternally inherited allele: the *IGF2* DMR0 (located between exons 2 and 3 of *IGF2*), the *IGF2* DMR2 (between exons 8 and 9) and the *H19* DMR, located 4 kb upstream of the transcription start of *H19*[15], which are all methylated to a level of 40-50% [10,16,17]. The methylation status of the *IGF2* DMR0 is more likely to be indicative of changes in *IGF2* transcription from the active allele given it has been suggested to possess promoter activity [18]. The *H19* DMR contains several recognition sites for the CCCTC binding factor (CTCF), which binds to these sites on the maternal allele to form a chromatin boundary which blocks *IGF2* transcription and promotes *H19* transcription from the maternal allele. CTCF cannot bind to the paternal allele, which is methylated at these sites, bringing enhancers downstream of *H19* in close proximity to the *IGF2* gene, enhancing its transcription, while *H19* is not expressed from the paternal allele. *The KCNQ10T1* ICR (also called KvDMR1) within intron 10 of *KCNQ1* is maternally methylated, and regulates the maternally expressed *CDKN1C* gene and the paternally expressed but untranslated protein KCNQ10T1 [19].

Two studies using bisulfite pyrosequencing to investigate the *IGF2* and *H19* DMRs in cord blood have found either no association [20] or a minor (1.6%) reduction in the methylation levels of the *IGF2* DMR0 [21] among infants born with low birthweight, but not necessarily at term. One previous study analyzing 19 children conceived by intracytoplasmic sperm injection and born small for gestational age (SGA), found that one child had a clear hypermethylation of *KCNQ10T1* with a methylation level of 74% [22]. Copy number variations in several growth related genes including *IGF2* but not *CDKN1C* or *KCNQ10T1* were examined in 100 SGA children with short stature, and although no *IGF2* deletions were found, two children were identified with IGF1R haploinsufficiency [23].

The aim of our study was therefore to investigate whether in the absence of assisted reproduction, common epigenetic variation (loss of methylation at the *IGF2*/*H19* ICR or gain of methylation at the *KCNQ10T1* ICR) or copy number variation (causing maternal duplication of the 11p15 region) are associated with the phenotype of SGA at term.

## Methods

### Participants

The Auckland Birthweight Collaborative (ABC) study has been previously described in detail [24]. In summary, from the original cohort of singleton babies without congenital abnormalities or assisted reproduction, born at term (37+ weeks gestation), and resident in Auckland in 1995–1997, all those of European ethnicity who were followed up at 11 years, and consented to analysis of DNA from a blood sample were included in this study. Written informed consent was obtained for genetic analysis, from the parent or guardian on behalf of the children enrolled in the study. The Northern X ethics committee approved the study. Samples from 153 children were available for this study, including 81 born SGA (≤10th percentile corrected for sex and gestational age).

### Molecular genetic analysis

DNA was extracted from blood samples using the DNA extraction kit from Qiagen. All samples were extracted at the same time. The methylation status of the *H19* and *KCNQ10T1* ICRs was investigated by multiplex ligation dependent probe amplification (MLPA) using the SALSA MLPA ME030-B1 BWS/SRS kit (lot 0208) purchased from MRC-Holland (Amsterdam, The Netherlands). This mix includes 26 probes specific for the BSW/SRS 11p15 region of which 11 probes contain a recognition site for the methylation-sensitive restriction endonuclease HhaI and can thereby provide information about the methylation status of the target sequence, including five within the *H19* DMR, and 5 within the *KCNQ10T1* DMR. The kit also contains additional 16 probes that are used as controls for copy number quantification and 4 control probes for complete HhaI digestion. MLPA analysis was performed according to the manufacturer’s protocol and has been previously described [25]. In brief, 100 ng of genomic DNA from each individual was denatured and hybridized overnight with the probe mixture supplied with the kit, and then, after dividing the sample into two aliquots, treated either with ligase alone or with ligase and HhaI. Polymerase chain reaction (PCR) reactions were performed with the primers supplied in the kit. Once microlitre of each PCR product was mixed with 0.5 μL of Genescan-Rox 500 size standard and 8.5 μL of deionised formamide, and 1 μL was injected into a 36 cm capillary (model 3130XL, Applied Biosystems Foster City, CA, USA). Electropherograms were analyzed using the Genescan software. Non-SGA samples were run as reference at the same time as SGA samples to ensure comparability and avoid any batch effects.

The quality of raw data was evaluated according to the manufacturer’s checklist (MRC Holland). For each sample the relative area under the peak was calculated using the Coffalyser software (version 9.4). First, data from digested and undigested probes were used to perform intra-sample normalization whereby the signal for each investigated probe was divided by the signal of all reference probes within the sample. The median of all ratios was used as a normalization constant for subsequent analysis. Next, the copy number in undigested samples was determined by dividing the normalisation constant of each probe of each patient sample by the average normalisation constant of all reference samples for that probe. The methylation level was obtained by dividing each normalization constant of the digested sample by the normalization constant of the corresponding undigested sample. Experiments were performed in triplicate and the mean of the standard deviations of the methylation ratios in each patient was 0.03 for the *H19* DMR and 0.02 for *KCNQ10T1*, respectively (ranges 0.02-0.06 and 0.01-0.03 respectively) as previously reported [26].

In addition, a subset of 58 samples (43 born SGA) was investigated using bisulfite pyrosequencing. 500 ng genomic DNA was treated with sodium bisulfite using the EpiTect Bisulfite conversion kit (Qiagen) and 50 ng of this DNA was used as a template for PCR amplification. Two DMRs were analyzed by pyrosequencing as previously described: the *IGF2* DMR0 (3 CpGs), and in the 3rd CTCF binding site of the *H19* DMR (11 CpGs) [27]. Accession numbers and nucleotide positions of each DMR, PCR primers and annealing temperatures as well as the size of PCR products have been previously described [28].

The MLPA methylation analysis investigated five sites within the *H19* DMR. HhaI ligation sites analyzed with reference to the Genbank sequence AF125183.1 were: 9769–9770 (*H19*a); 9605–9606 (*H19*b), 9449–9450 (*H19*c), 9144–9145 (*H19*d), 8691–8692 (*H19*e). The five HhaI ligation sites within the *KCNQ10T1* DMR were with respect to Genank AJ006345.1: 254373–254374 (*KCNQ10T1*a); 254444–254445 (*KCNQ10T1*b), 254890–254891 (*KCNQ10T1*c), 255226–255227 (*KCNQ10T1*d), 333007–333008 (*KCNQ10T1*e). Pyrosequencing was used to analyse 11 CpG sites within CTCF3 of the *H19* DMR and 3 CpGs within the *IGF2* DMR0.

### Statistical analysis

The characteristics of the population were described as mean and SD for birthweight. We used t-tests to assess the difference in the mean methylation levels of the *H19* DMR, *KCNQ10T1* DMR, *IGF2* DMR0 and CTCF3 between the SGA and AGA groups. Statistical significance was defined at the 5% level.

## Results

No copy number variations within any of the 11p15 genes involved in BWS or SRS were detected. One child was found to have markedly reduced methylation at all probe locations within the *KCNQ10T1* DMR, (reported separately in [29]), and was excluded from this analysis.

There was no significant difference in the methylation levels within the *H19* DMR or *KCNQ10T1* DMR between children born SGA compared to non-SGA using MLPA (Table 1). When we analysed the subset who had pyrosequencing analysis, there was no significant difference in the mean methylation levels at CTCF3. However, the DNA methylation level at CpG1 of the *IGF2* DMR0 was statistically significant lower (2.7%) among cildren born SGA compared to those born AGA (SGA mean [sd] 44.1 [3.8] vs 47.2 [3.8], p = 0.009). However, no difference was observed at the other two CpG sites (Table 1).

**Table 1:** Methylation by SGA status

**MLPA**	**SGA (N = 80) % mean methylation (standard deviation)**	**AGA (N = 72) % mean methylation (standard deviation)**	**T-statistic (p value)**
*H19* ICR (1)	56.3 (7.3)	57.3 (7.6)	−0.84 (0.40)
*H19* ICR (2)	62.0 (6.6)	61.9 (5.7)	0.11 (0.92)
*H19* ICR (3)	62.7 (6.1)	61.9 (6.6)	0.75 (0.45)
*H19* ICR (4)	68.2 (9.1)	67.3 (7.5)	0.69 (0.49)
*H19* ICR (5)	87.2 (7.7)	85.8 (6.7)	1.10 (0.27)
Average	66.4 (4.7)	66.3 (3.7)	0.17 (0.87)
*KCNQ10T1 *(1)	58.3 (7.5)	60.0 (7.5)	−1.41 (0.16)
*KCNQ10T1* (2)	54.1 (6.7)	53.9 (6.9)	0.16 (0.87)
*KCNQ10T1* (3)	54.7 (7.6)	55.9 (7.1)	−0.99 (0.33)
*KCNQ10T1* (4)	61.7 (9.1)	63.2 (8.9)	−1.01 (0.31)
*KCNQ10T1* (5)	66.7 (11.0)	67.4 (14.6)	−0.32 (0.75)*
Average	57.2 (5.4)	58.2 (5.6)	−1.17 (0.24)
**Pyrosequencing** (CTCF3 *H19* DMR)	SGA (N = 43)	AGA (N = 15)	
CpG2	41.6 (3.1)	40.6 (3.1)	1.03 (0.31)
CpG3	50.4 (7.4)	49.9 (11.1)	0.17 (0.87)*
CpG4	60.1 (5.1)	61.6 (10.3)	−0.56 (0.58)*
CpG5	40.6 (3.3)	40.1 (3.1)	0.48 (0.63)
CpG6	46.2 (2.7)	45.5 (3.0)	0.82 (0.42)
CpG7	45.1 (2.2)	45.2 (2.5)	−0.10 (0.92)
CpG8	44.6 (2.4)	44.3 (2.9)	0.41 (0.68)
CpG9	71.4 (6.5)	71.4 (9.6)	0.01 (0.99)
CpG10	43.3 (3.7)	41.8 (2.9)	1.42 (0.16)
CpG11	42.6 (2.6)	42.0 (2.5)	0.82 (0.41)
CpG12	38.5 (2.5)	38.2 (3.2)	0.38 (0.71)
Average	47.3 (2.8)	47.3 (3.4)	−0.05 (0.96)
*IGF2*DMR0			
CpG1	44.1 (3.8)	47.2 (3.8)	*−2.69 (0.0094)*
CpG2	55.3 (2.8)	56.9 (4.3)	−1.37 (0.19)
CpG3	61.7 (3.7)	64.5 (10.2)	−1.04 (0.31)*
Average	53.6 (2.5)	56.2 (5.1)	−1.90 (0.075)

*Variances not equal.

**Figure 1:**
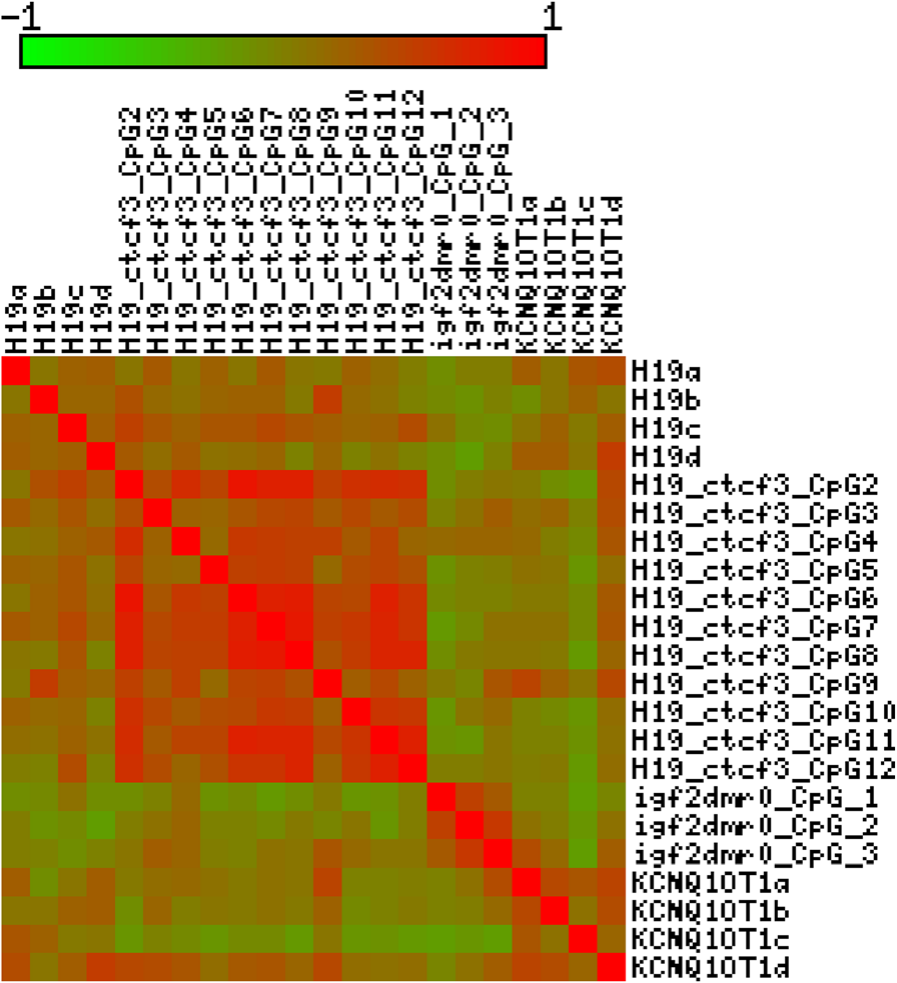
Graphical representation of the correlation between methylation sites.

Figure 1 shows the correlation of all the methylation sites examined. DNA methylation of the five different HhaI ligation sites within *KCNQ10T1* was poorly correlated (range −0.23 to 0.46). DNA methylation of the 5 HhaI ligation sites within the *H19* DMR was also poorly correlated (range −0.17 to 0.25). Correlation between the 11 different CpG sites of the CTCF3 site within the *H19* DMR region interrogated by pyrosequencing was moderate (r = 0.49 range (0.16 to 0.83)). The correlation between the 3 CpG sites within the *IGF2* DMR0 was relatively weak (mean r = 0.30 (range −0.02 to 0.54)).

## Discussion

In the present study, we examined whether SGA at term was associated with copy number variation or altered methylation levels at two imprinted genetic loci (*H19* DMR*, IGF2* DMR0 and *KCNQ10T1* DMR) known to be associated with SRS or BWS on chromosome 11p15.5. We found a statistically significant 2.7% reduction in methylation at *IGF2* DMR0 in peripheral blood leucocytes collected from 11 year old children born SGA compared to those born AGA. This association would not have reached the level of statistical significance after correction for multiple testing, and we cannot exclude that this result occurred by chance. However, our result is consistent with a previous study which reported 1.6% lower methylation at the same *IGF2* DMR0 using cord blood DNA from infants born with low birthweight [21]. We did not find any copy number variation.

We used a commercially available MLPA kit, which enabled simultaneous interrogation of copy number and methylation status of multiple loci relevant to BWS/SRS. We observed little correlation between the non-contiguous methylation sites interrogated either by MLPA or pyrosequencing methods. Although we did not expect any correlation between *H19* DMR*, IGF2* DMR0 and the more centromeric *KCNQ10T1* DMR which are two distinct imprinting domains, we also observed little correlation between the 5 MLPA sites within the *H19* DMR. We observed little correlation between the *H19* sites interrogated by MLPA, and either the CTCF3 or the *IGF2* DMR0 CpGs interrogated by pyrosequencing.

Although the MLPA method for detecting BWS and SRS abnormalities has been demonstrated to have good intra-assay and inter-assay reproducability [26], this method may have limited sensitivity to detect minor variation in methylation levels. However, selective hypomethylation for either the *H19* DMR or the *IGF2* DMR0 has been previously reported in patients with SRS [30]. Selective hypomethylation of the *IGF2* DMR0 but not the *H19* DMR has previously been associated with lower birthweight [21], and the converse for maternal folic acid intake [20].

## Conclusion

This study confirms minor hypomethylation of the *IGF2* DMR0 in peripheral blood leucocytes of children born SGA at term, which is not reflected at other imprinted sites within the *IGF2/H19* domain. Copy number variation within the 11p15 BWS/SRS region does not seem to be an important cause of non-syndromic SGA at term.

## Abbreviations

BWS: Beckwith Wiedemann Syndrome; CTCF: CCCTC-binding factor; DMR: Differentially methylated region; ICR: Imprinting control region; MLPA: Multiplex ligation dependent probe amplification; SGA: Small for gestational age; SRS: Silver-Russell syndrome.

## Competing interests

All authors have no conflicts of interest to declare.

## Authors’ contributions

RM conceived the study, interpreted the data and drafted the manuscript. JT acquired the pyrosequencing data and assisted with data interpretation. JMDT performed the statistical analysis of all genetic data. EM contributed to interpretation of data. All authors were involved in revising the manuscript. All authors read and approved the final manuscript.

## Pre-publication history

The pre-publication history for this paper can be accessed here:

http://www.biomedcentral.com/1471-2350/15/67/prepub
